# The Ontology of Biological and Clinical Statistics (OBCS) for standardized and reproducible statistical analysis

**DOI:** 10.1186/s13326-016-0100-2

**Published:** 2016-09-14

**Authors:** Jie Zheng, Marcelline R. Harris, Anna Maria Masci, Yu Lin, Alfred Hero, Barry Smith, Yongqun He

**Affiliations:** 1 Department of Genetics, University of Pennsylvania Perelman School of Medicine, Philadelphia, PA 19104 USA; 2 Division of Systems Leadership and Effectiveness Science, University of Michigan School of Nursing, Ann Arbor, MI 48109 USA; 3 Department of Biostatistics and Bioinformatics, Duke Medical Center, Duke University, Durham, NC 27710 USA; 4 Department of Microbiology and Immunology, Unit for Laboratory Animal Medicine, University of Michigan Medical School, Ann Arbor, MI 48109 USA; 5 Department of Electrical Engineering and Computer Science, Department of Biomedical Engineering, and Department of Statistics, Michigan Institute of Data Science, University of Michigan, Ann Arbor, MI 48109 USA; 6 Department of Philosophy and National Center for Ontological Research, University at Buffalo, Buffalo, NY 14203 USA

**Keywords:** OBCS, Biological statistics, Clinical outcomes statistics, Standardization, Statistical analysis, Data integration

## Abstract

**Background:**

Statistics play a critical role in biological and clinical research. However, most reports of scientific results in the published literature make it difficult for the reader to reproduce the statistical analyses performed in achieving those results because they provide inadequate documentation of the statistical tests and algorithms applied. The Ontology of Biological and Clinical Statistics (OBCS) is put forward here as a step towards solving this problem.

**Results:**

The terms in OBCS including ‘data collection’, ‘data transformation in statistics’, ‘data visualization’, ‘statistical data analysis’, and ‘drawing a conclusion based on data’, cover the major types of statistical processes used in basic biological research and clinical outcome studies. OBCS is aligned with the Basic Formal Ontology (BFO) and extends the Ontology of Biomedical Investigations (OBI), an OBO (Open Biological and Biomedical Ontologies) Foundry ontology supported by over 20 research communities. Currently, OBCS comprehends 878 terms, representing 20 BFO classes, 403 OBI classes, 229 OBCS specific classes, and 122 classes imported from ten other OBO ontologies.

We discuss two examples illustrating how the ontology is being applied. In the first (biological) use case, we describe how OBCS was applied to represent the high throughput microarray data analysis of immunological transcriptional profiles in human subjects vaccinated with an influenza vaccine. In the second (clinical outcomes) use case, we applied OBCS to represent the processing of electronic health care data to determine the associations between hospital staffing levels and patient mortality. Our case studies were designed to show how OBCS can be used for the consistent representation of statistical analysis pipelines under two different research paradigms. Other ongoing projects using OBCS for statistical data processing are also discussed.

The OBCS source code and documentation are available at: https://github.com/obcs/obcs.

**Conclusions:**

The Ontology of Biological and Clinical Statistics (OBCS) is a community-based open source ontology in the domain of biological and clinical statistics. OBCS is a timely ontology that represents statistics-related terms and their relations in a rigorous fashion, facilitates standard data analysis and integration, and supports reproducible biological and clinical research.

## Background

The movement to advance reproducibility of research advocates the use of open data, standard operating procedures, and reproducibility of methods used in both computation [1] and statistics [2]. To support such reproducibility it is imperative that standard metadata formats be used to describe how results were generated. Consider, for example, the case of ImmPort (the Immunology Database and Analysis Portal; https://immport.niaid.nih.gov/), which is the world’s largest repository of public-domain de-identified clinical trial data related to immunology [3, 4]. All data derived from clinical trials funded by the Division of Allergy, Immunology and Transplantation (DAIT) of the National Institute of Allergy and Infectious Diseases are required to be published on the ImmPort portal. In addition, the ImmPort portal includes data obtained from the work of the Human Immunology Project Consortium (HIPC, www.immuneprofiling.org/) as well as relevant data from a number of external sources such as the Gates Foundation. ImmPort currently contains data sets from 211 studies with 34,801 subjects and 1054 experiments, including complete clinical and mechanistic study data all of which are publicly available for download in a deidentified form. To facilitate data import and processing, ImmPort has created templates for data representation and documented standard operating procedures for working with imported data. To facilitate discovery and usability of these data to external users, and also to address the goal of reproducibility, ImmPort is seeking wherever possible to draw on publicly available ontologies such as the Cell Ontology (CL) [5] and the Protein Ontology (PRO) [6] as sources for the terms used in these templates.

If the information-driven research documented in resources like ImmPort is to be reproducible, however, then the research results contained in the ImmPort and similar repositories must also be annotated using ontology terms which specify the protocols and methods used in data generation and analysis. The OBI has been created to this end, and the OBCS follows in the footsteps of OBI by providing terms and formal definitions representing the statistical methods used in biological and clinical research. OBCS will thereby not merely allow researchers to reproduce statistical analyses in order to validate the conclusions reached by study authors but also allow new possibilities for discovery of and for comparison between studies. We believe that it will also serve as impetus for the creation of new sorts of software tools supporting more advanced use of statistics in complex analysis and meta-analysis of large and heterogeneous data sets.

Ontologies are human- and computer-interpretable representations of the types of entities existing in specific scientific domains and of the relations between these types. Since the creation of the first version of the Gene Ontology (GO) in 1998 [7], many influential ontology resources have been created, most recently following the principles of the Open Biological and Biomedical Ontologies (OBO) Foundry [8]. Ontologies built in accordance with OBO Foundry principles are designed to allow not only consistent classification, comparison and integration across heterogeneous datasets, but also automatic reasoning with the data annotated with their terms. This is achieved in part through the adoption of Basic Formal Ontology (BFO) [9] as a common top-level ontology, and also through employment of a common set of logically defined relations. OBI is a prominent example of ontology that aligns with BFO. OBI provides a set of logically defined terms covering a broad range of investigation processes, experimental conditions, types of equipment and documentation, and data analysis methods performed on data generated from the experiments [10, 11]. OBO ontologies were initially successful in the representation of model organism research data and have subsequently been influential in health-related data standardization and processing [12]. Ontobee, the default OBO ontology linked server, incorporates over 100 OBO ontologies and provides the facility to track how ontology terms are reused in multiple ontologies [13].

OBCS has its origin in a 2010 study of the ANOVA (ANalysis Of VAriance) meta-analysis of vaccine protection assays [14], which led to the addition into OBI of statistical terms related to ANOVA and survival rate analysis. Later, an OBI statistics branch was generated to identify and fill gaps in OBI’s representation of statistics [15]. The OBCS resulted directly from these efforts. OBCS extends OBI, and the two ontologies share overlapping development groups.

## Methods

### OBCS development

OBCS is a community-based ontology of statistical tools and methods used in biological and clinical investigations that follows OBO Foundry principles in providing the possibility for enhanced representation and analysis of data generated through complex statistical procedures.

OBCS is expressed using the W3C standard Web Ontology Language (OWL2) (http://www.w3.org/TR/owl-guide/). The meta-data schema of OBCS is implemented using OWL annotation properties defined in the Information Artifact Ontology (IAO, http://purl.obolibrary.org/obo/iao), which is widely used by OBO Foundry and other ontologies (https://github.com/information-artifact-ontology/IAO/wiki/OntologyMetadata). In what follows, we use single quotes to represent terms from ontologies (including OBCS), and italics to represent object properties (also known as relations) such as *is_a* and *part_of*. The Protégé OWL editor (http://protege.stanford.edu/) was used for ontology editing and the Hermit reasoner (http://hermit-reasoner.com/) for consistency checking and inferencing. OBCS-specific terms were generated with IDs using the prefix “OBCS_” followed by auto-generated seven-digit numbers.

OBCS is released under Creative Commons Attribution (CC BY) 3.0 License, and the OBCS source code is freely available at: https://github.com/obcs/obcs and also on the Ontobee [13] (http://www.ontobee.org/ontology/OBCS) and NCBO BioPortal (http://purl.bioontology.org/ontology/OBCS) websites. The summary information of ontology terms in OBCS based on term types and resources can be found here: http://www.ontobee.org/ontostat/OBCS.

The RDF triples for the OBCS ontology have been saved in the He group Triple store [13], which allows easy retrieval of related OBCS contents using Semantic Web SPARQL technology. OBCS can be queried from the Ontobee’s SPARQL query endpoint (http://www.ontobee.org/sparql) [13].

A combination of top-down and bottom-up methods was used in OBCS development. The top-down approach was initiated by extending OBCS from the latest version of OBI (http://purl.obolibrary.org/obo/obi/2015-12-07/obi.owl) using the BFO 2.0 classes-only version (http://purl.obolibrary.org/obo/bfo/2014-05-03/classes-only.owl) and the middle tier ontology IAO (http://purl.obolibrary.org/obo/iao/2015-02-23/iao.owl) [11]. OBCS also reuses ontological models (as described in the Results section) developed in OBI and IAO. The remaining parts of the ontology were then populated on the basis of a survey of statistics workflows, which led to the identification of a number of statistics-related terms not included in other ontologies. These terms were supplemented through the prototype statistics data analysis workflow proposed in [14] and through the study of specific use cases described below.

This bottom-up strategy was combined with a top-down approach involving definition terms through downward migration from higher-level ontology classes. For example, the Robust Multi-Array Average (RMA) normalization is a commonly used microarray data normalization method [16] and the corresponding term ‘robust multi-array average normalization’ (OBCS_0000140) was generated as a subclass of OBI ‘normalization data transformation’. Populating the ontology in this way provided a simple strategy for creating definitions of the terms introduced using the method of specific difference [17] since the genus (and its definition) were inherited from OBI it was necessary for purposes of OBCS to supply only the differentia.

### Reusing existing ontology resources

OBCS imports the subset of OBI consisting of all statistics-related terms and associated parent terms using the Ontodog tool [18]. The Excel input data used for this purpose is available at: https://github.com/obcs/obcs/raw/master/docs/OBI_statisitics_subset.xlsx. To ensure valid reasoning, Ontodog was set to import all the terms used in the expression of the ontological axioms relating to each imported OBI term. For example, when Ontodog fetched the OBI term ‘log-log curve fitting’, the axiom:

‘log-log curve fitting’: ‘*achieves planned objective*’ some ‘curve fitting objective’ was also retrieved, together with the terms ‘*achieves planned objective*’ and ‘*curve fitting objective*’.

To eliminate redundancy and ensure orthogonality, terms already defined in OBO Foundry ontologies were reused in accordance with the Minimum Information to Reference an External Ontology Term (MIREOT) guideline [19]. OntoFox, a software program implementing and extending MIREOT [20], was used to extract individual terms from external ontologies using this strategy.

### Driving use cases for OBCS development

The first of two use cases driving OBCS development concerns a study of the systems biology of influenza vaccination described in [21], relating to a transcription profiling by array experiment which has as its objective the identification of the gene expression profiles in human study subjects after influenza vaccine administration. Human blood specimens were used in this study, so that both biological and clinical domains were involved. The second use case concerns the study of clinical outcomes of nursing services data with the aim of investigating statistical associations between variable levels of nurse staffing and inpatient mortality [22]. Using observational data collected from a clinical setting, a Cox proportional hazards model estimation was conducted to draw the conclusion that understaffed shifts were significantly associated with increased inpatient mortality [22]. ‘Transcription profiling by array experiment’ and ‘Cox proportional hazards model estimation’ are among the OBCS terms deriving from these use cases.

## Results

### OBCS overview and high level hierarchy structure

The latest release of OBCS contains a total of 878 terms, including 780 classes, 42 object properties, 25 annotation properties, and 6 datatype properties. Among these 780 classes, 229 classes are unique to OBCS, 403 were imported from OBI. The remaining terms were imported from various OBO ontologies, such as BFO (20 classes), IAO (51 classes), the Statistics Ontology (STATO) (http://stato-ontology.org/) (36 classes), the Phenotypic Quality Ontology (PATO) (10 classes) [23] (Table 1).

**Table 1:** Summary of ontology terms in OBCS as of June 4, 2016

Ontology Names	Classes	Object properties	Datatype properties	Annotation properties	Instance	Total
OBCS	229	2	0	1	1	233
OBI (Ontology for Biomedical Investigations)	403	9	2	3	4	421
IAO (Information Artifact Ontology)	51	8	4	13	18	94
STATO (Statistics Ontology)	36	0	0	0	0	36
BFO (Basic Formal Ontology)	20	6	0	2	0	28
PATO (Phenotypic Quality Ontology)	10	0	0	0	0	10
RO (Relation Ontology)	0	17	0	1	1	19
Other ontologies^a^	31	0	0	4	1	42
Total	780	42	6	25	25	878

^a^the name and statistics of other ontologies used in OBCS can be found on the Ontobee website: http://www.ontobee.org/ontostat/OBCS

**Fig. 1:**
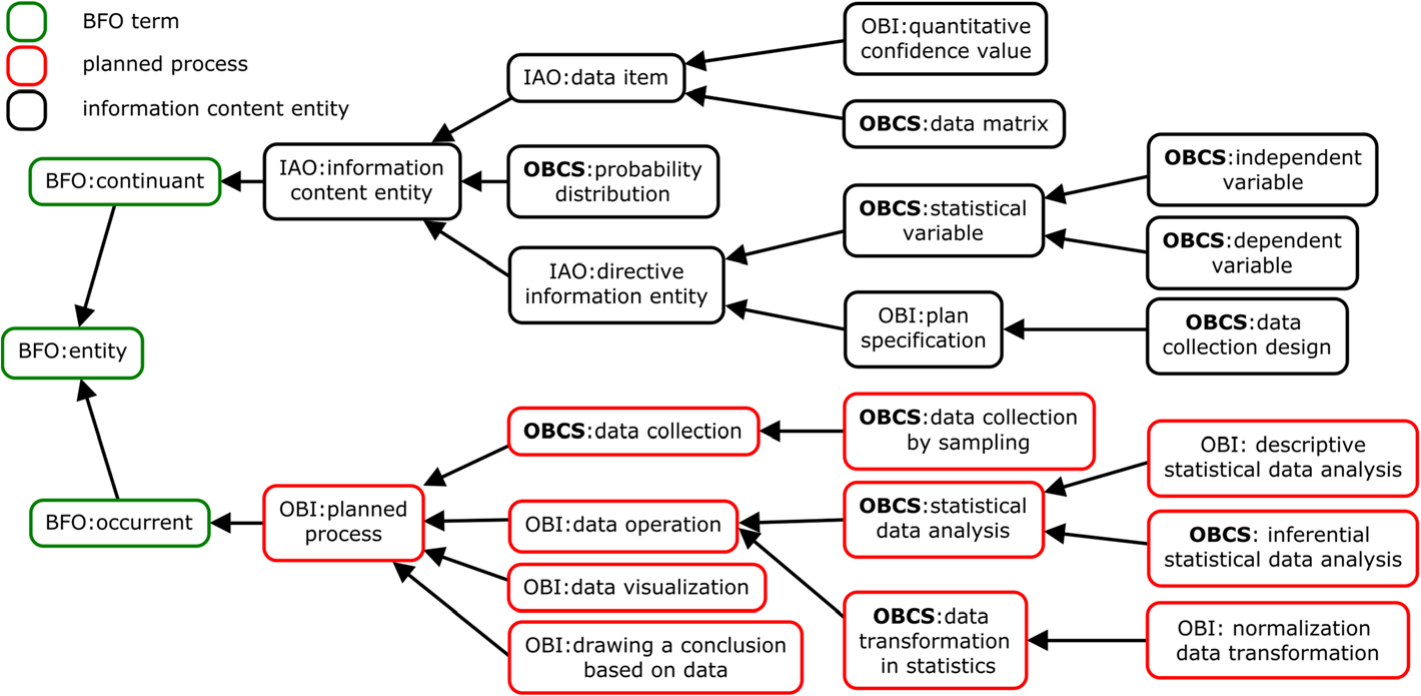
The top level OBCS hierarchical structure and key ontology terms. The terms shown in boxes with the prefix “OBCS:” in bold font are OBCS-specific terms, and the other terms are imported from existing ontologies including BFO, IAO and OBI

Figure 1 illustrates the top-level hierarchical structure and some key ontology terms of OBCS, showing terms from both the ‘continuant’ and ‘occurrent’ branches of BFO [9]. The continuant branch represents entities (e.g., ‘material entity’) which endure through time; the ‘occurrent’ branch represents entities such as ‘process’ which occur in time. Many key continuant terms in OBCS are classified under IAO’s ‘information content entity’, including: ‘data item’, ‘probability distribution’, as well as ‘directive information entities’ such as ‘protocols’ and ‘algorithms’ (Fig. 1). OBCS includes 178 subclasses under the branch of IAO data item (IAO_0000027).

Major occurrent terms in OBCS relate to different types of planned process, including: ‘data collection’, ‘data operation’, ‘data visualization’, and ‘drawing a conclusion based on data’. The term ‘data operation’ (OBI_0200000; a new alternative label for the imported OBI term ‘data transformation’) is defined as “A planned process that produces output data from input data”. ‘Data operation’ satisfies two logical axioms:

*has_specified_input only/some* ‘data item’

*has_specified_output only/some* ‘data item’

Two important child terms of ‘data operation’ are: ‘data transformation in statistics’ and ‘statistical data analysis’ (Fig. 1). A ‘data transformation in statistics’ converts a ‘data set’ to another ‘data set’ by applying a deterministic mathematic function to each data item in the input data set. For example, the OBI term ‘logarithmic transformation’ (OBI_0200094) represents the kind of process that transforms input data to output data by applying a logarithm function with a given base. In this case, the data transformation process *concretizes realize* the mathematical function. A key subclass under ‘data transformation in statistics’ is ‘normalization data transformation’, which includes various normalization processes such as ‘robust multi-array average normalization’ (RMA) [16].

### General design pattern used in the OBCS representation of statistical studies

**Fig. 2:**
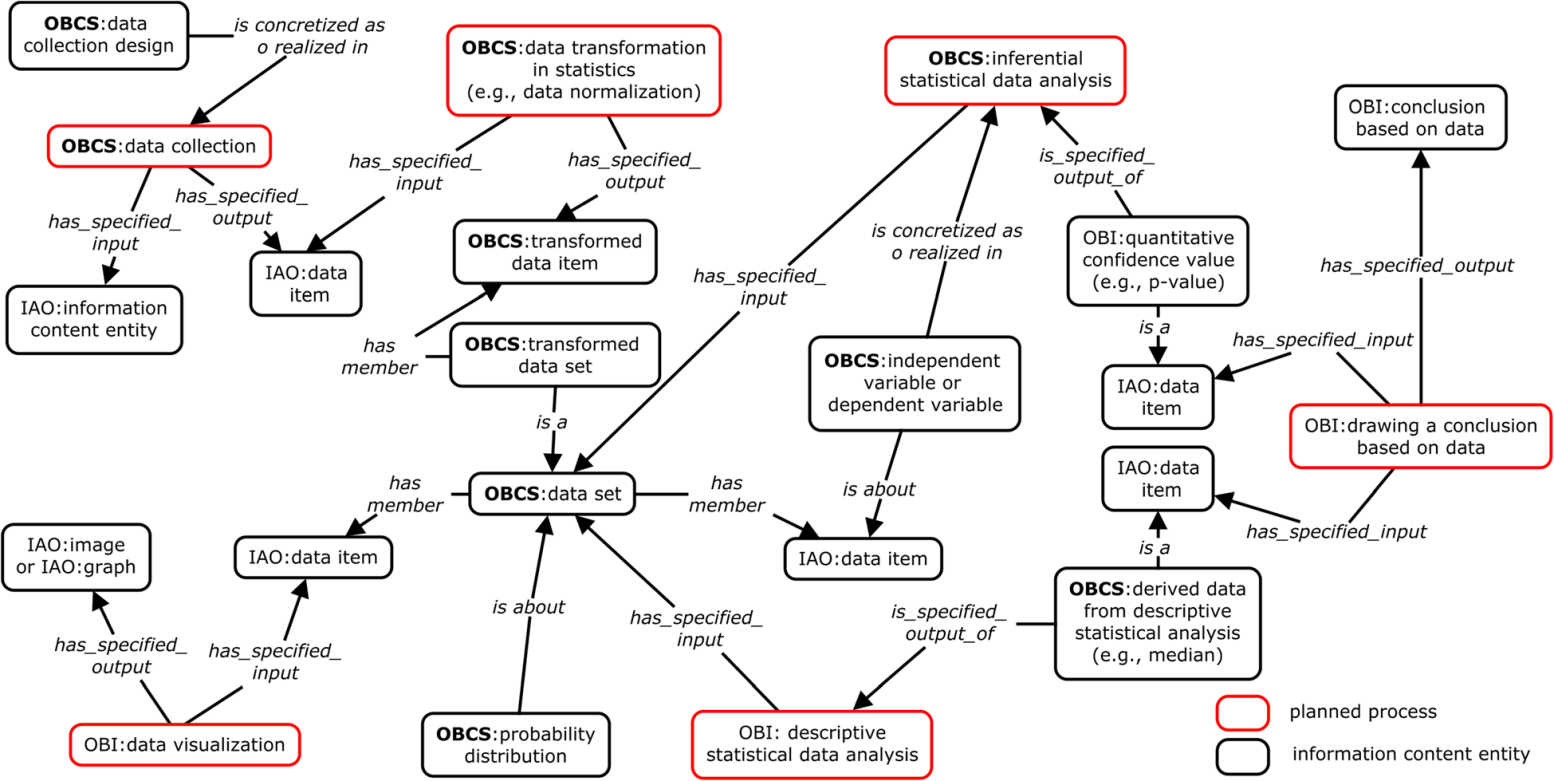
Semantic representation of statistical data analysis studies using OBCS. The boxes highlighted in red represent key planned processes in OBCS, and the terms in black boxes represent different information content entities

**Fig. 3:**
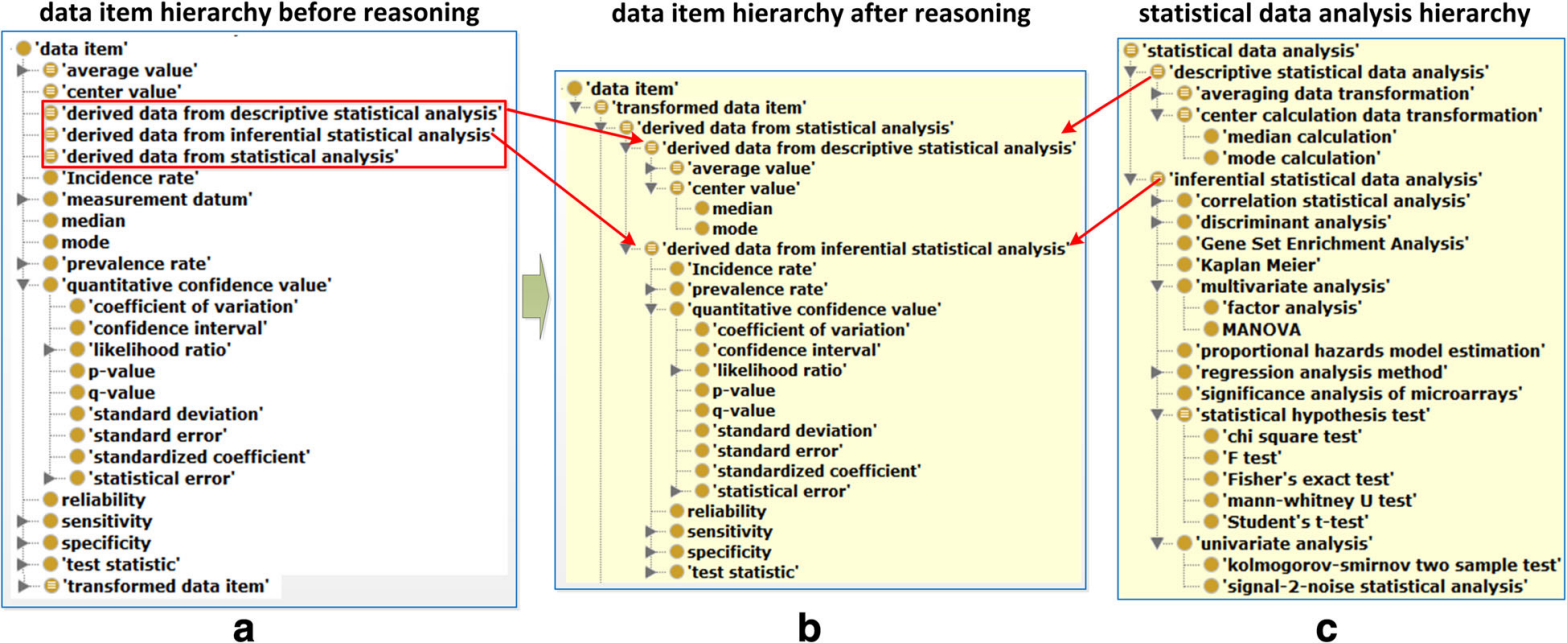
Illustration of selected OBCS terms under ‘data item’ and ‘statistical data analysis’ and their hierarchies. **a** Illustration of part of the asserted hierarchical structure for the OBCS branch of ‘data item’. Note that there is no subclass under ‘derived data from statistical analysis’. **b** The inferred hierarchical structure after using the reasoner HermiT 1.3.8. After the reasoning, the ‘derived data from statistical analysis’ has two direct subclasses ‘derived data from descriptive statistical analysis’ and ‘derived data from inferential statistical analysis’. **c** Illustration of a part of the ‘statistical data analysis’ branch in OBCS. Note that many OBCS terms under these branches are not shown, and these selected terms are used for demonstration. These screenshots came from the OBCS display using the Protégé OWL editor

OBCS is designed to represent all aspects of a statistical study. A general statistical study workflow is represented in Fig. 2, which shows five major processes including: ‘data collection’, ‘data transformation in statistics’, ‘statistical data analysis’, ‘data visualization’, and ‘drawing a conclusion based on data’. These are all subtypes of OBI ‘planned process’ (Fig. 1) which comprehends two major subtypes of data item (Fig. 3), namely: ‘measurement datum’ and ‘derived data from statistical analysis’. The latter is further divided into: ‘derived data from descriptive statistical analysis’ (e.g., ‘median’ and ‘mode’) and ‘derived data from inferential statistical analysis’ (e.g., *p*-value).

OBCS defines many different types of data directly under ‘data item’, and then provides logical axioms that can be used to infer a data type under a particular class based on a statistical data analysis (Fig. 3). For example, ‘derived data from inferential statistical analysis’ is defined by an equivalence class axiom as:

‘data item’ and (*is_specified_output_of* some ‘inferential statistical data analysis’)

Given this equivalence class, every ‘data item’ subclass (e.g., ‘specificity’) having the axiom of *is_specified_output_of* some ‘inferential statistical data analysis’ will be inferred to be a subclass of ‘derived data from inferential statistical analysis’.

**Fig. 4:**
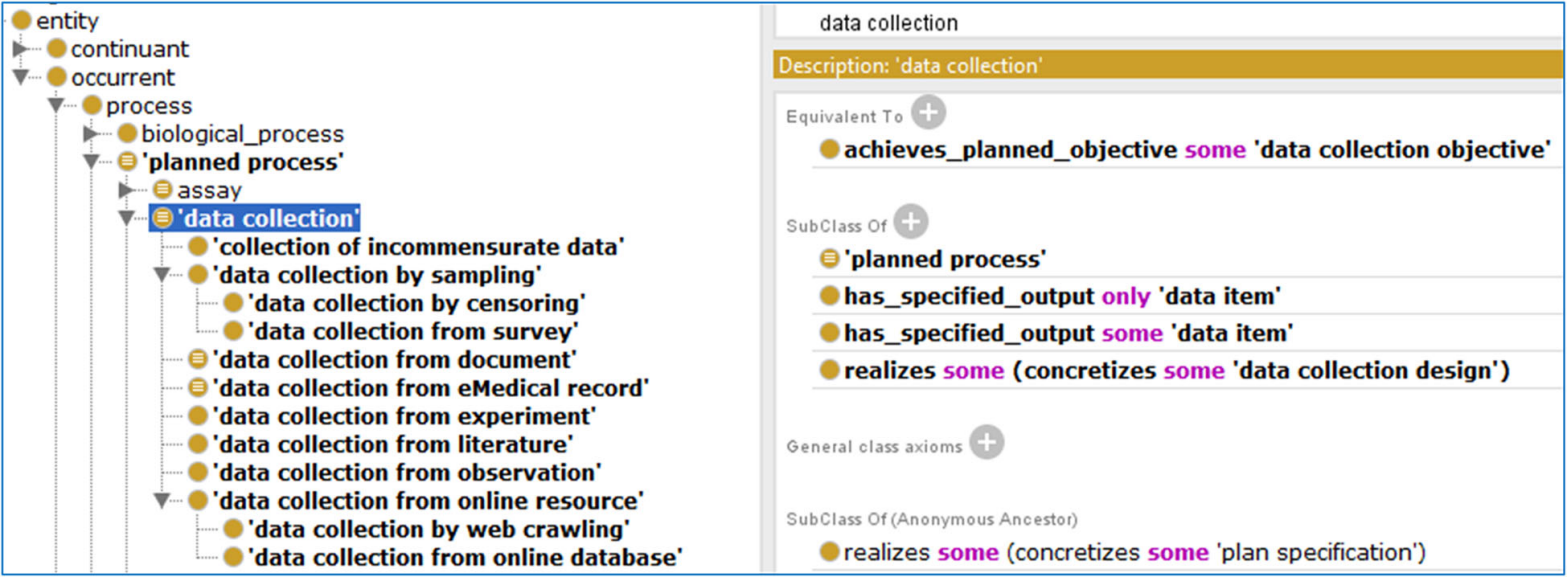
Illustration of logical axioms and subclasses of ‘data collection’ in OBCS. This is a screenshot of Protégé OWL editor display of OBCS. As shown here, this term is defined by an equivalent axiom, four subclass axioms, and one inherited subclass axiom. There are 12 subclasses under ‘data collection’

Next we will introduce how OBCS represents each of the five major processes. Figure 4 represents the major parts of the OBCS ‘data collection’ branch, which comprehends 12 subclasses corresponding to different approaches to data collection, such as from experiment, literature, observation, by online extraction, or by sampling. Online extraction can be performed from online databases or through a web crawler. Sampling can be achieved through survey or censoring. Data collection may face difficulties of different sorts. For example, data fields may be incommensurable, data may be missing, incompatibilities may arise due to different types of study design, and we may have only partial information concerning data provenance, and so forth. To address these factors OBCS includes terms such as ‘generation of missing data’, which represents a planned process that generates possible values of missing data. OBCS also includes the term ‘processing incompatible data’ that represents a data transformation process that attempts to transforms incompatible data into data that are compatible. Different methods can be used to support these processes.

**Fig. 5:**
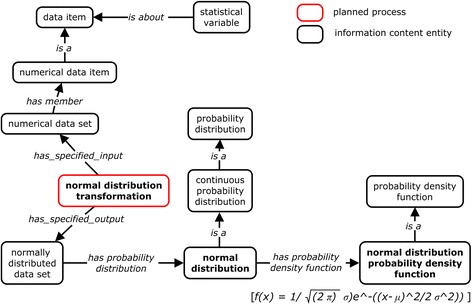
OBCS modeling of statistical terms related to normal distribution. The normal (or Gaussian) distribution is a continuous probability distribution of a numerical data set that follows the normal distribution probability density function. The formula of the density function is at the bottom of the figure and included in OBCS as a ‘mathematical formula’ annotation. A normal distribution transformation is able to transform a data set to normally distributed data set

After data are collected, the data often need to be reorganized or processed by different data transformation processes that transform the data into a format suitable for ‘statistical data analysis’. A typical method is to transform a list of data by associating data items with probability values yielding one or other type of probability distribution, for example, ‘normal (or called Gaussian) distribution’ (Fig. 5). Such a distribution follows a specific ‘probability density function’, a term that is also included in OBCS. ‘Normalization data transformation’ is a commonly used ‘data transformation in statistics’ that adjusts values measured on different scales to a notionally common scale and makes variables comparable to each other. OBCS defines 34 different types of normalization methods. Other data processing methods applied before ‘statistical data analysis’ – for example ‘permutation’, ‘sorting’, or ‘data partitioning’ – are also represented in OBCS.

Figure 1 represents are two types of statistical data analysis methods called ‘descriptive’ and ‘inferential’, respectively. A ‘descriptive statistical data analysis’ quantitatively summarizes a feature of a collection of data. It includes the application of many statistical operations that summarize a data set in terms of its ‘arithmetic mean’, ‘standard deviation’, ‘empirical probability distribution’, and so on. An ‘inferential statistical data analysis’, in contrast, infers properties of a collection of data through analysis of data. An ‘inferential statistical analysis’ includes testing hypotheses and deriving estimates. These methods can be performed on multiple data sets and thereby generate a ‘*p*-value’, ‘R-squared value’, ‘likelihood ratio’, or other ‘quantitative confidence value’ (Fig. 3c). In total, OBCS now includes 12 types of ‘descriptive statistical analysis’ methods and 92 types of ‘inferential statistical analysis’ methods.

OBCS defines a ‘statistical variable’, including ‘independent’ and ‘dependent variable’, as a ‘directive information entity’ that is about a ‘data item’ and can only be realized in a ‘statistical analysis’. Without a ‘statistical analysis’, a statistical variable does not exist. For example, a clinical study may have collected data about age, sex, weight, and whether diabetes. Age data item is about age quality. A statistician can specify that an independent variable (for instance an age-independent variable) *is about* an age data item and a dependent variable *is about* whether a given individual has diabetes, and then test whether age significantly affects the likelihood of the occurrence of diabetes in a human population. The age data item *is about* the age quality of a human subject. A scalar measurement datum includes two parts: data value and data unit. If a human subject is 20 years old, the age data item can be represented as:

*((‘has value’ value 20) and (‘has measurement unit label’ some ‘year’))* and *‘is about’ some age*.

The age data item will vary from subject to subject, which is the reason why we can get an age data set in a study. For example, if a clinical study includes three human subjects whose ages are 20, 40, and 50 years, then the three age data items form an age data set.

One or multiple data sets can be visualized as an ‘image’ or ‘graph’ by performing a process of ‘data visualization’ (Fig. 2). Two logical axioms are defined for ‘data visualization’:

*has_specified_input* only/some ‘data item’

*has_specified_output* only/some (‘graph’ or ‘image’)

Currently, OBCS includes four subclasses of ‘data visualization’: ‘clustered data visualization’, ‘gene list visualization’, ‘classified data visualization’, and ‘background corrected data visualization’. To support data visualization, OBCS imports 25 terms from the ‘graph’ branch in the STATO ontology.

Based on the results of a statistic data analysis, we can ‘draw a conclusion based on data’ (Fig. 2). The ‘descriptive statistical analysis’ results (such as ‘median’ and ‘mode’) describe the features of a ‘data set’. The result of an ‘inferential statistical data analysis’, such as a ‘*p*-value’ (a type of ‘quantitative confidence value’), is used to help us to ‘draw a conclusion’ either accepting or rejecting a ‘hypothesis’. One important ‘quantitative confidence value’ is ‘R-squared value’, which is often used for analyzing prediction problems. The class ‘draw a conclusion based on data’ also includes subclasses corresponding to different sorts of biological feature conclusions, such as ‘comparative phenotypic assessment’ and ‘assigning gene property based on phenotypic assessment’.

Next we focus on the OBCS modeling of the two use cases introduced in the Methods section above.

### OBCS statistical representation of a systems vaccinology use case

**Fig. 6:**
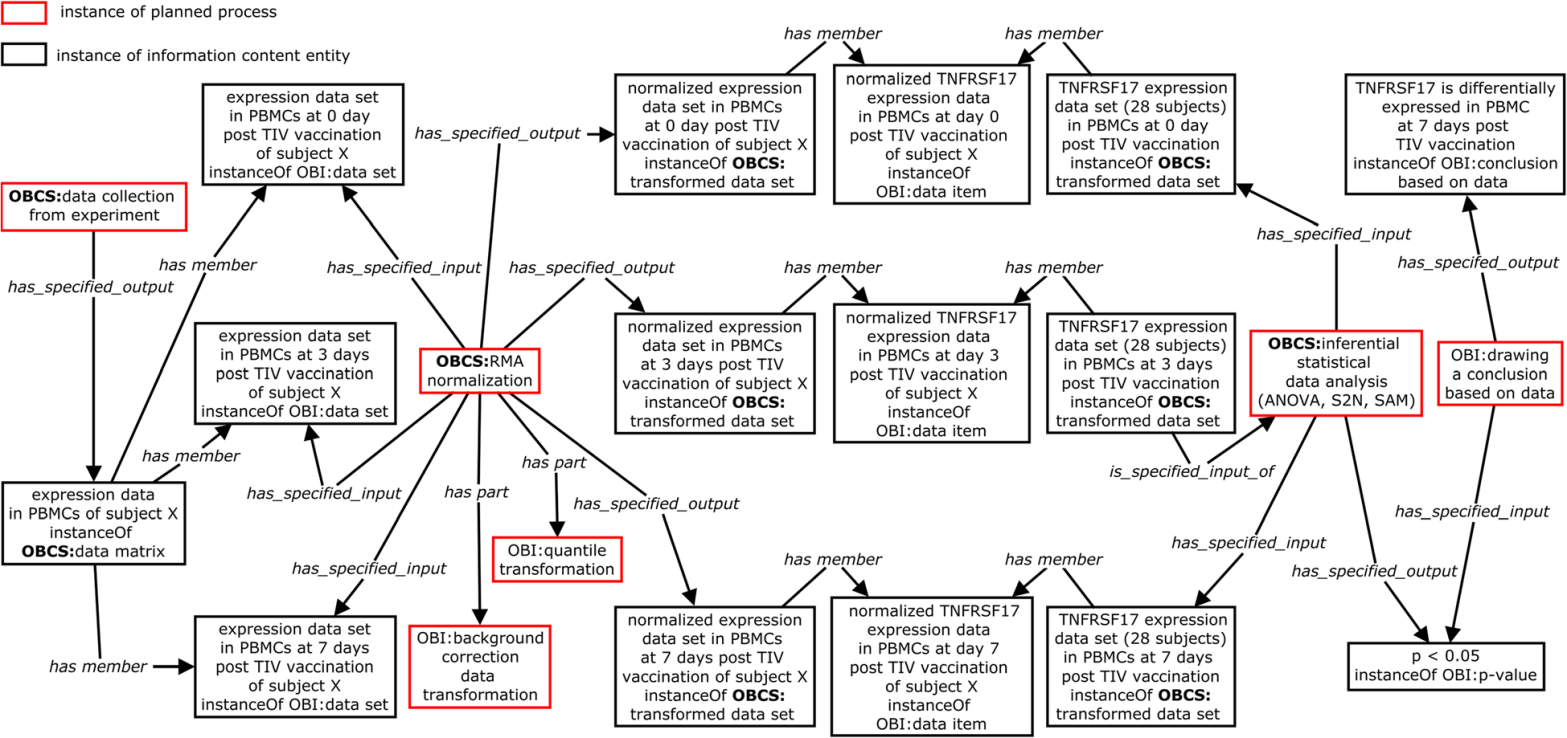
Ontological representation of an influenza microarray study. In this example, each of 28 human subjects was vaccinated once with an influenza vaccine [21]. At day 0, 3, and 7 post vaccination, human blood samples were extracted, peripheral blood mononuclear cell (PBMCs) were isolated from the blood samples, and RNAs were prepared from PBMCs. An Affymetrix microarray experiment was then conducted using the RNA samples. After RMA data normalization, the gene expression data from different groups (separated on the basis of time) were used for three types of statistical tests (ANOVA, S2N and SAM). All the boxes represent instances, labelled by the class names of these instances. All the relations are italicized

The first use case, which is of a sort typically found in high throughput biomarker analysis, is a study selected from the field of systems vaccinology [21]. Each of the twenty eight enrolled human subjects was vaccinated once with Fluarix, a trivalent inactivated influenza vaccine (TIV). At days 0 (baseline), 3 and 7 post vaccination, ‘blood specimens’ were collected from human subjects and from these samples peripheral blood mononuclear cells (PBMCs) were prepared. ‘RNA extracts’ were then prepared from the PBMCs and used in a ‘transcription profiling assay’ examining expression of a large number of genes using Affymetrix microarray technology. The basic investigation procedure can be presented using OBI. For the statistical steps however the additional resources of OBCS are needed. To illustrate how OBCS is used for annotation or tagging of statistics workflows we single out one human subject (subject X) (Fig. 6). The initial statistical data analysis step is ‘data collection from experiment’. Such a ‘data collection’ process ‘has specified output’ the raw gene expression data at different time points post vaccination. After the raw data collection, the gene expression results for individual genes such as TNFRSF17 (tumor necrosis factor receptor superfamily, member 17) at days 0 (baseline) and 7 post vaccination were normalized using the ‘Robust Multi-array Average (RMA)’ method [16]. The ‘RMA’ (OBCS_0000140) statistical method has the following two asserted axioms (Fig. 2b):

‘RMA’: *has_part some* ‘background correction data transformation’

‘RMA’: *has_part some* ‘quantile transformation’

The above ‘RMA data normalization’ process ensures that all Affymetrix microarray data from the study can be analyzed. Normalized gene expression values for any given gene (for example TNFRSF17) across all subjects at different time points can then be used in a range of statistical tests, including ‘ANOVA’, ‘signal-2-noise analysis (S2N)’ and ‘significance analysis of microarrays (SAM)’. The ‘*p*-values’ for these tests are then reported, the ‘*p*-value’ of <0.05 obtained for all 3 tests indicating that a null hypothesis that the two groups (baseline Day 0 vs Day 7) have the equal means of gene expression intensities can be rejected. From this we can ‘draw a conclusion’ that the gene in question is significantly regulated in study subjects consequent to influenza vaccination (Fig. 6).

Note that for simplicity some important experimental factors (such as ‘sex’, ‘age’, and microarray format) and statistical results (for example ‘fold change’) are not represented in the Figure. These factors can be represented using OBO Foundry-related ontologies such as PATO and the Experimental Factor Ontology (EFO) [24]. Representation of these factors can be incorporated in a statistical analysis using the OBCS approach. The Figure provides, we believe, a good illustration of how OBI and OBCS are used to annotate the sorts of biostatistics studies commonly found in high throughput molecular assay analysis and biomarker analysis.

For users to explore and better understand how OBCS can help biomedical data annotation and analysis, we provided an example data set and supplemental materials available for downloading at https://github.com/obcs/obcs/wiki/OBCS-example. The example contains an Ontodog-generated OBCS subset including all the terms, relations, and axioms introduced in the Fig. 6 use case. We created instances of the OBCS subset classes to present transcriptional expressions of 3 genes (TNFRST17, MAPK1, and CNOT2) in the PMBCs collected from three individuals at day 0, 3, and 7 after an administration of TIV. This example shows how data items (e.g., gene expression intensities) can be grouped together to form a data set (e.g., the set of expression intensities of the genes in a microarray chip for one sample), and how data sets themselves can be grouped together to form a data matrix, which is data set of higher order (for example a set of gene expression data obtained from applying multiple microarray chips to multiple samples). As shown in Fig. 6, planned processes link the data sets collected from experiments to the conclusion based on data drawn from statistical data analysis.

### OBCS representation of a clinical outcomes research use case

**Fig. 7:**
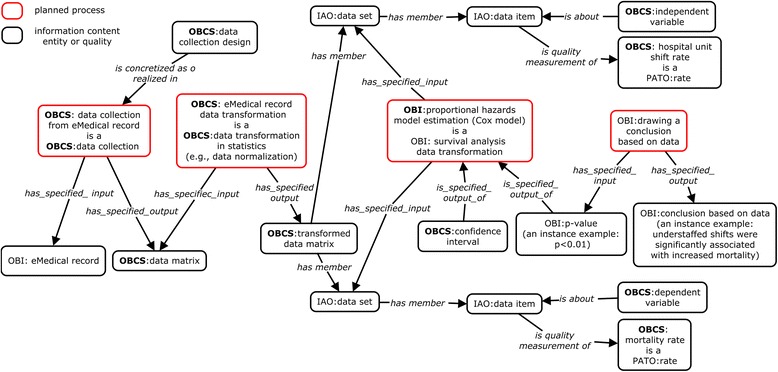
Ontological representation of a clinical study. This use case study analyzed the effects of variable levels of nurse staffing on inpatient mortality [22]. The data was obtained from eMedical records and then transformed. Cox proportional hazards model estimation (a type of survival analysis) was performed to identify the effect of hospital unit shift rates (independent variable) on the patient mortality rate (dependent variable). A *p*-value of <0.01 was used to draw a conclusion of the statistical significance between these two variables

The second use case is focused on medical informatics analysis in clinical research. In a study of clinical outcomes of nursing services [22], data were obtained from ‘electronic medical records’ (EMR) and transformed to data with standard measurement units. This study examined the effect of variable levels of nurse staffing on inpatient mortality. The ‘statistical analysis’ of these variables is represented ontologically in OBCS (Fig. 7). On a shift-by-shift basis, the unit on which each patient was located was identified and unit characteristics and staffing data for the shift were merged with outcomes-relevant patient data. This process resulted in 3,227,457 separate records, with information for each patient relating to each shift during the period in which they were hospitalized. The records included measures of patient-level and unit-level characteristics, nurse staffing, other shift-specific measures, and patient mortality. In this study, the initial statistical data analysis step is ‘data collection from an observation’, rather than from an experiment. ‘Covariates’ were constructed to adjust for factors not controlled in the study and yet still affecting the ‘dependent variable’, which is in this case mortality. Such a process ‘has specified output’ the difference between targeted and actual staffing levels at different time points during hospital stay. The relative risk of increased mortality was then estimated using ‘Cox proportional hazards models’. Reported statistical results included ‘*p*-value’, ‘mean’, ‘standard deviation’, ‘confidence interval’, and ‘standardized mortality ratio’. The obtained ‘*p*-value’ of <0.05 indicates that a null hypothesis of equal average of ‘mortality ratios’ between those with and without exposure to understaffed shifts was rejected. Therefore, we can ‘draw a conclusion’ that understaffed shifts were significantly associated with increased mortality (Fig. 7).

### OBCS data query

**Fig. 8:**
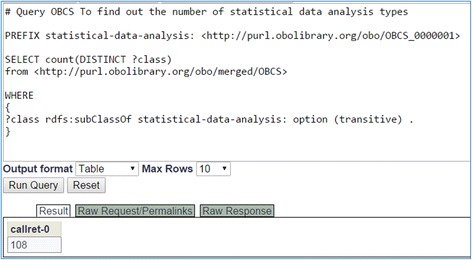
SPARQL query of the number of statistic data analysis methods in OBCS. This SPARQL query was performed using the Ontobee SPARQL query website (http://www.ontobee.org/sparql/). This query found 108 statistical data analysis methods in OBCS. These terms are all under the OBCS term ‘statistical data analysis’ (OBCS_0000001)

OBCS can be queried using SPARQL, a Resource Description Framework (RDF) query language for retrieving ontology data stored in the RDF format [25]. Figure 8 shows how a simple SPARQL query can be used to identify the number of methods under the OBCS class ‘statistical data analysis’ (OBCS_0000001). As shown in Figs. 1 and 3, there are two types of statistical data analysis: inferential and descriptive. The SPARQL query can recursively search all the layers of the two branches and identify the total number of subclasses in each branch.

## Discussion

There is a critical need to standardize data representation, including standardization and formal representation of statistical methods. In this paper, we have introduced Ontology of Biological and Clinical Statistics (OBCS), focusing on the introduction of high level statistics terms in OBCS and on how OBCS can be used in combination with OBI for the ontological representation of statistics-related biological and clinical data processing. OBCS provides a timely source of statistical terms and semantics in various areas of biological and clinical statistics. OBCS provides a consensus-based rigorously curated representation of the steps involved in statistics pipelines in different domains in biological and clinical fields, thereby supporting reproducibility of research.

The current OBCS development team is composed of researchers from a number of complementary backgrounds. Jie Zheng (PhD) is an experienced ontology developer and biomedical researcher. Marcelline Harris (PhD, RN) is a domain expert in clinical statistics. Alfred Hero (PhD, with appointment in the Department of Statistics at the University of Michigan) is a domain expert in statistics and biostatistics. Anna Maria Masci (PhD) is an immunologist who is well trained in ontology. Dr Yu Lin (MD, PhD) is experienced in both clinical and biomedical informatics and ontology development. Barry Smith (PhD) is a co-creator of the BFO ontology and of the OBO Foundry. Yongqun He (DVM, PhD) is an ontology developer and a domain expert in vaccine and immunology research as well as computer science.

OBCS and OBI are closely related. OBCS extends OBI by focusing on data collection, normalization and statistical analysis performed on data. Some core terms in OBCS are taken from OBI but the term coverage in OBCS includes terms relating not only to data generated through experiments but also to data from other resources such as survey studies, text mining, clinical observations, online databases, and so on. While many statistical data analysis methods and related terms, including ‘ANOVA’ and ‘*p*-value’, are used quite generally, there are also statistical methods and terms that are applied only in certain specific domains. The RMA [16] and GSEA (gene set enrichment analysis) [26] methods, for example, are used only in relation to biological data. Since OBCS originated from and inherits its coverage domain from OBI, it places its emphasis on biomedical statistics. However, OBCS’s coverage goes beyond that of OBI, since it contains terms relating to statistical methods commonly used in clinical fields. The major purpose of OBCS is to provide a standardized representation of statistical processes, methods and algorithms across both the biological and clinical science. OBCS can serve as an integrative metadata platform to support statistical data representation, analysis, and data integration and facilitate statistical validation, reproducibility, and discovery of published research involving statistical analysis.

Several ontologies have been developed that contain terms related to statistics [27–29]. Above all, the STATO (http://stato-ontology.org/) was recently announced. While OBCS is focused on biological and clinical statistics, STATO aims to cover a broader scope and to include also natural science domains outside the life sciences. OBCS and STATO thus focus on different aspects of statistics. After STATO was officially released, we reused STATO terms in OBCS wherever applicable. We have imported many terms with a focus especially on the graph branch of STATO. To deal with the residual sets of terms common to both OBCS and STATO, we added the STATO-OBCS mapping information using the oboInOwl:‘database_cross_reference’ annotation property in OBCS. STATO terms contain many logical axioms, which can be used for generating queries such as those listed at http://stato-ontology.org/. Such implementations are very helpful will be incorporated within OBCS wherever possible. On the other hand, the major driving use case for OBCS is to serve annotation of biomedical data in a way that promotes reproducibility – and eventual automation – of statistical data analyses for the whole sets of biomedical experimental data [30]. As a result, OBCS focuses more than STATO on providing detailed data analysis pipelines, as illustrated in Figs. 2, 5, 6, and 7, to support systematic statistical data analyses. We are collaborating with STATO to achieve a consensus division of development effort in the future.

The OntoDM ontology [27], developed with a focus on data mining, also has some statistical components, as does the Hypothesis and Law Ontology (HELO), which focuses on representing probabilistic scientific knowledge and hypothesis evaluation [28]. In addition, the Ontology of Clinical Research (OCRe) incorporates some statistics terms used in clinical studies [29]. However, OCRe, in contrast to all the aforementioned ontologies, does not provide definitions for its terms, and it is not aligned with BFO or OBI. After careful comparison and examination, we found that none of these ontologies focuses on the sort of comprehensive representations of biological and clinical statistics that meets our needs in formally representing the statistical tools and methods used in data collection, organization, analysis, presentation and interpretation. OBCS is the first ontology that systematically represents the five major processes in statistical studies (Fig. 2), and lays out general design patterns for representing statistical distributions (*e.g.*, normal distribution as shown in Fig. 5) and related terms. OBCS also includes many statistics terms unavailable in other ontologies.

Our two use cases lie at opposite ends of the translational science continuum from T0 (basic biomedical research) to T4 (translation to population). These use cases demonstrate the usage of OBCS in basic and translational biomedical research. We are developing OBCS applications addressing other points on this continuum, including T1 (basic to clinical translation), T2 (demonstrating efficacy), T3 (translation to practice) [31, 32].

In addition to the two use cases detailed in this manuscript, OBCS is currently being used for the standardized representation and annotation of genomics data analysis in the Beta Cell Genomics Database (http://www.betacell.org/gbco/). The OBCS design pattern and strategies are consistent with previous research of using the combination of OBI [10] and the Vaccine Ontology [33, 34] to support the statistical meta-analysis of variables contributing to the effects of protective immunity [14, 35]. OBCS is being tested in the work of the ImmPort project team for standard data representation and statistical data analysis, and it is being evaluated also for its ability to support statistical software tools such as RImmPort [4] and Python for Population Genomics (PyPop; http://www.pypop.org/) developed to promote reproducible and automated analysis of biological and clinical data. Since OBI, VO, PATO, and other OBO ontologies provide many terms to represent features of biological and clinical studies, OBCS can use the corresponding terms when representing the corresponding experimental variables (such as ‘vaccine’, ‘gender’, ‘age’, ‘mortality’) in a statistical analysis.

In the future, OBCS will be further developed to include new statistical methods and inference procedures, support data integration, and be applied to more resources. The OBCS development is driven primarily by use cases. Our current use cases have been focused on vaccinology, immunology, flow cytometry, microarray, and clinical nursing scenarios. Many more statistics-specific questions and cases will still be generated and studied in these areas. In addition to standard statistical data representation and integration, OBCS will be useful also in supporting more consistent extraction of data, thereby allowing new kinds of search (for example: for all data derived using a specific type of analysis or a specific type of variable). Many statistical methods have been implemented in different software programs such as many statistical programs available in BioConductor [36] and RImmPort [4]. We plan to relate OBCS statistical analysis methods to corresponding software programs by reusing the Software Ontology (SWO) terms [37]. Such linkage between OBCS statistical methods and software programs can support standard statistical method representation and software integration, reproducible data analysis, and interoperable communications between different software programs. We believe that biomedical and clinical databases and software programs targeting big data analysis will benefit considerably from the standardized definitions and logical representations of statistics terms and relations [30] of the sort which OBCS provides.

## Conclusion

The Ontology of Biological and Clinical Statistics (OBCS) is a community-based open source ontology in the domain of biological and clinical statistics. We presented the rationale, history, scope, contents, top level hierarchy, and a general design pattern of OBCS. Second, we provided detailed accounts of the main branches of OBCS, including ‘data item’, ‘statistical data analysis’, and ‘data collection’. Third, the OBCS approach to statistical terms related to normal distribution is presented, and it is shown how this approach can be generalized to other statistical distributions. Fourth, two OBCS use case are studied and presented, demonstrating how OBCS can be applied to the ontological representation of real statistical studies. Lastly, a SPARQL query example (Fig. 8) is provided to demonstrate how to quickly query OBCS information stored in an RDF triple store. Overall, we believe that OBCS is a timely ontology able to represent statistics-related terms and their relations in a rigorous fashion, facilitate standard data analysis and integration, and support reproducible biological and clinical research.

## References

[1] Stodden V, Guo P, Ma Z (2013). Toward Reproducible Computational Research: An Empirical Analysis of Data and Code Policy Adoption by Journals. PLoS One.

[2] Stodden V (2014). The reproducible research movement in statistics. Stat J IAOS.

[3] Bhattacharya S, Andorf S, Gomes L, Dunn P, Schaefer H, Pontius J, Berger P, Desborough V, Smith T, Campbell J (2014). ImmPort: disseminating data to the public for the future of immunology. Immunol Res.

[4] Shankar RD, Andorf S, Bhattacharya S, Wiser JA, Butte AJ: RImmPort: enabling ready-for-analysis immunology research data. In: Proceedings of the 5th ACM Conference on Bioinformatics, Computational Biology, and Health Informatics: September 22–25 2013; Washington, D.C.

[5] Meehan TF, Masci AM, Abdulla A, Cowell LG, Blake JA, Mungall CJ, Diehl AD (2011). Logical development of the cell ontology. BMC Bioinformatics.

[6] Natale DA, Arighi CN, Blake JA, Bult CJ, Christie KR, Cowart J, D’Eustachio P, Diehl AD, Drabkin HJ, Helfer O (2014). Protein Ontology: a controlled structured network of protein entities. Nucleic Acids Res.

[7] Ashburner M, Ball CA, Blake JA, Botstein D, Butler H, Cherry JM, Davis AP, Dolinski K, Dwight SS, Eppig JT (2000). Gene ontology: tool for the unification of biology. The Gene Ontology Consortium. Nat Genet.

[8] Smith B, Ashburner M, Rosse C, Bard J, Bug W, Ceusters W, Goldberg LJ, Eilbeck K, Ireland A, Mungall CJ (2007). The OBO Foundry: coordinated evolution of ontologies to support biomedical data integration. Nat Biotechnol.

[9] Grenon P, Smith B (2004). SNAP and SPAN: Towards Dynamic Spatial Ontology. Spat Cogn Comput.

[10] Brinkman RR, Courtot M, Derom D, Fostel JM, He Y, Lord P, Malone J, Parkinson H, Peters B, Rocca-Serra P (2010). Modeling biomedical experimental processes with OBI. J Biomed Semant.

[11] Bandrowski A, Brinkman R, Brochhausen M, Brush MH, Bug B, Chibucos MC, Clancy K, Courtot M, Derom D, Dumontier M (2016). The Ontology for Biomedical Investigations. PLoS One.

[12] Schulz S, Balkanyi L, Cornet R, Bodenreider O (2013). From Concept Representations to Ontologies: A Paradigm Shift in Health Informatics?. Healthcare Inf Res.

[13] Xiang Z, Mungall C, Ruttenberg A, He Y: Ontobee: A linked data server and browser for ontology terms. In: The 2nd International Conference on Biomedical Ontologies (ICBO): 2011; Buffalo, NY, USA. CEUR Workshop Proceedings; 2013: Pages 279–281.

[14] He Y, Xiang Z, Todd T, Courtot M, Brinkman RR, Zheng J, Stoeckert CJ, Jr., Malone J, Rocca-Serra P, Sansone SA et al: Ontology representation and ANOVA analysis of vaccine protection investigation. In: Bio-Ontologies 2010: Semantic Applications in Life Sciences: July 11–13 2010; Boston, MA, USA. CEUR Workshop Proceedings: Pages 1–8 [http://ceur-ws.org/Vol-754/he_krmed2010.pdf]. Accessed 12 Sept 2016.

[15] OBI: Ann Arbor 2012 OBI Workshop presentations: https://svn.code.sf.net/p/obi/code/trunk/docs/presentations/OBI%20workshop%20May%202012%20Ann%20Arbor/. In.; 2012. Accessed 12 Sept 2016.

[16] Irizarry RA, Hobbs B, Collin F, Beazer-Barclay YD, Antonellis KJ, Scherf U, Speed TP (2003). Exploration, normalization, and summaries of high density oligonucleotide array probe level data. Biostatistics.

[17] Arp R, Smith B, Spear AD (2015). Building Ontologies Using Basic Formal Ontology.

[18] Zheng J, Xiang Z, Stoeckert CJ, He Y (2014). Ontodog: a web-based ontology community view generation tool. Bioinformatics.

[19] Courtot M, Gibson F, Lister A, Malone J, Schober D, Brinkman R, Ruttenberg A (2011). MIREOT: the Minimum Information to Reference an External Ontology Term. Appl Ontol.

[20] Xiang Z, Courtot M, Brinkman RR, Ruttenberg A, He Y (2010). OntoFox: web-based support for ontology reuse. BMC Res Notes.

[21] Nakaya HI, Wrammert J, Lee EK, Racioppi L, Marie-Kunze S, Haining WN, Means AR, Kasturi SP, Khan N, Li GM (2011). Systems biology of vaccination for seasonal influenza in humans. Nat Immunol.

[22] Needleman J, Buerhaus P, Pankratz VS, Leibson CL, Stevens SR, Harris M (2011). Nurse staffing and inpatient hospital mortality. N Engl J Med.

[23] PATO - Phenotypic Quality Ontology [https://github.com/pato-ontology/pato/]. Accessed 12 Sept 2016.

[24] Malone J, Holloway E, Adamusiak T, Kapushesky M, Zheng J, Kolesnikov N, Zhukova A, Brazma A, Parkinson H (2010). Modeling sample variables with an Experimental Factor Ontology. Bioinformatics.

[25] Harris S, Seaborne A: SPARQL 1.1 Query Language, W3C Recommendation 21 March 2013. 2013: URL: http://www.w3.org/TR/sparql11-query/. Accessed 14 Aug 2013.

[26] Subramanian A, Tamayo P, Mootha VK, Mukherjee S, Ebert BL, Gillette MA, Paulovich A, Pomeroy SL, Golub TR, Lander ES (2005). Gene set enrichment analysis: A knowledge-based approach for interpreting genome-wide expression profiles. Proc Natl Acad Sci U S A.

[27] Panov P, Dzeroski S, Soldatova LN: OntoDM: An Ontology of Data Mining. In: IEEE International Conference on Data Mining Workshops (ICDMW ’08) 2008; Pisa. 752–760.

[28] Soldatova LN, Rzhetsky A, De Grave K, King RD (2013). Representation of probabilistic scientific knowledge. J Biomed Semant.

[29] Sim I, Tu SW, Carini S, Lehmann HP, Pollock BH, Peleg M, Wittkowski KM: The Ontology of Clinical Research (OCRe): An informatics foundation for the science of clinical research. J Biomed inform 2014;52:78–91. http://www.ncbi.nlm.nih.gov/pubmed/24239612 10.1016/j.jbi.2013.11.002 PMC4019723 24239612

[30] Russ TA, Ramakrishnan C, Hovy EH, Bota M, Burns GA (2011). Knowledge engineering tools for reasoning with scientific observations and interpretations: a neural connectivity use case. BMC Bioinformatics.

[31] Westfall JM, Mold J, Fagnan L (2007). Practice-based research--“Blue Highways” on the NIH roadmap. JAMA.

[32] Kon AA (2008). The Clinical and Translational Science Award (CTSA) Consortium and the translational research model. Am J Bioeth.

[33] He Y, Cowell L, Diehl AD, Mobley HL, Peters B, Ruttenberg A, Scheuermann RH, Brinkman RR, Courtot M, Mungall C, et al. VO: Vaccine Ontology. In: The 1st International Conference on Biomedical Ontology (ICBO-2009): July 24–26 2009; Buffalo, NY, USA. Nature Precedings: http://precedings.nature.com/documents/3552/version/1; 2009.

[34] Ozgur A, Xiang Z, Radev DR, He Y (2011). Mining of vaccine-associated IFN-gamma gene interaction networks using the Vaccine Ontology. J Biomed Semant.

[35] Todd TE, Tibi O, Lin Y, Sayers S, Bronner DN, Xiang Z, He Y (2013). Meta-analysis of variables affecting mouse protection efficacy of whole organism Brucella vaccines and vaccine candidates. BMC Bioinformatics.

[36] Gentleman RC, Carey VJ, Bates DM, Bolstad B, Dettling M, Dudoit S, Ellis B, Gautier L, Ge Y, Gentry J (2004). Bioconductor: open software development for computational biology and bioinformatics. Genome Biol.

[37] Malone J, Brown A, Lister AL, Ison J, Hull D, Parkinson H, Stevens R (2014). The Software Ontology (SWO): a resource for reproducibility in biomedical data analysis, curation and digital preservation. J Biomed Semant.

